# Monitoring for micronutrient deficiency after bariatric surgery—what is the risk?

**DOI:** 10.1038/s41430-023-01318-3

**Published:** 2023-08-07

**Authors:** Carrie-Anne Lewis, Emma J. Osland, Susan de Jersey, George Hopkins, Matthew Seymour, Lindsey Webb, Mark D. Chatfield, Ingrid J. Hickman

**Affiliations:** 1 Department of Nutrition and Dietetics, Royal Brisbane and Women’s Hospital, Herston, QLD Australia; 2 The University of Queensland, Greater Brisbane Clinical School, Faculty of Medicine, Brisbane, QLD Australia; 3 The University of Queensland, School of Human Movements and Nutrition Science, Brisbane, QLD Australia; 4 The University of Queensland, Centre for Health Services Research, Faculty of Medicine, Brisbane, QLD Australia; 5 Department of Surgery, Royal Brisbane and Women’s Hospital, Brisbane, QLD Australia; 6 Department of Endocrinology, Royal Brisbane and Women’s Hospital, Brisbane, QLD Australia; 7 Department of Nutrition and Dietetics, Princess Alexandra Hospital, Brisbane, QLD Australia; 8 The University of Queensland, Centre for Clinical Research, Faculty of Medicine, Brisbane, Australia

**Keywords:** Nutrition, Pathology

## Abstract

**Background:**

Bariatric surgery may increase the risk of micronutrient deficiencies; however, confounders including preoperative deficiency, supplementation and inflammation are rarely considered.

**Objective:**

To examine the impact of bariatric surgeries, supplementation and inflammation on micronutrient deficiency.

**Setting:**

Two public hospitals, Australia.

**Methods:**

Participants were recruited to an observational study monitoring biochemical micronutrient outcomes, supplementation dose, inflammation and glycaemic control, pre-operatively and at 1–3, 6 and 12 months after gastric bypass (GB; Roux-en-Y Gastric Bypass and Single Anastomosis Gastric Bypass; *N* = 66) or sleeve gastrectomy (SG; *N* = 144). Participant retention at 12 months was 81%.

**Results:**

Pre-operative micronutrient deficiency was common, for vitamin D (29–30%), iron (13–22%) and selenium (39% GB cohort). Supplement intake increased after surgery; however, dose was <50% of target for most nutrients. After SG, folate was vulnerable to deficiency at 6 months (OR 13 [95% CI 2, 84]; *p* = 0.007), with folic acid supplementation being independently associated with reduced risk. Within 1–3 months of GB, three nutrients had higher deficiency rates compared to pre-operative levels; vitamin B1 (21% vs. 6%, *p* < 0.01), vitamin A (21% vs. 3%, *p* < 0.01) and selenium (59% vs. 39%, *p* < 0.05). Vitamin B1 deficiency was independently associated with surgery and inflammation, selenium deficiency with improved glycaemic control after surgery and inflammation, whilst vitamin A deficiency was associated with inflammation only.

**Conclusion:**

In the setting of prophylactic post-surgical micronutrient prescription, few nutrients are at risk of de novo deficiency. Although micronutrient supplementation and monitoring remains important, rationalising high-frequency biochemical testing protocols in the first year after surgery may be warranted.

## Introduction

Bariatric surgery reduces individual burden of disease by effectively treating obesity-related comorbidities [1]. However, the gastrointestinal tract resection and/or diversion may increase risk of micronutrient deficiencies that could increase an individuals’ burden of disease [2, 3]. International practice guidelines recognise a heightened risk and attempt to mitigate this by recommending prophylactic supplementation for a broad range of micronutrients postoperatively, often at high doses, and frequent routine biochemical micronutrient monitoring [2, 3]. However, the certainty of evidence underpinning the frequency of biochemical micronutrient monitoring and appropriate dose of micronutrient supplementation is poor [4, 5]. Given these recommendations come at a financial and personal cost to individuals and the healthcare system [6], further investigation is warranted to better understand the risk of micronutrient deficiency, and models of care designed to mitigate this risk.

Limitations within current literature describing micronutrient deficiency risk after surgery include a poor description of post-operative adherence to micronutrient supplements and the impact of supplements on reported biochemical micronutrient markers [4, 5] and emerging evidence suggests that in some patients, pre-existing micronutrient deficiencies may already be apparent prior to surgery [7, 8]. In addition, common factors known to confound the interpretation of biochemical micronutrient levels in obesity, such as presence of systemic inflammation [9] poor glycaemic control [10–12] and use of metformin [13, 14], have not been adequately interrogated in the context of micronutrient deficiencies after bariatric surgery.

The aim of this study was to examine the impact of bariatric surgery on micronutrient deficiency in the first postoperative year and investigate the relationship between both serum micronutrient levels and deficiency rates with supplementation dose, presence of inflammation and medication use within 1–3, 6 and 12 months of surgery, to inform models of care for biochemical micronutrient testing and prophylactic supplementation.

## Materials and methods

### Patient selection

This prospective observational study recruited consecutive participants from two tertiary hospitals in Brisbane, Australia, between November 2016 to July 2018. Included participants met international criteria for bariatric surgery and underwent a Sleeve Gastrectomy (SG) or Gastric Bypass (Roux-en-Y Gastric Bypass (RYGB) or Single-Anastomosis Gastric Bypass (SAGB); collectively referred to as Gastric Bypass [GB]) [2]. Exclusion criteria included <18 years of age, pregnant, unable or unwilling to give consent, undergoing primary gastric band or revisional bariatric surgery.

### Surgical technique

Sleeve gastrectomy was performed by six surgeons across two facilities using the same technique. The stomach was divided along a 36Fr bougie, 5–7 cm proximal to the pylorus. RYGB was performed by four surgeons at one facility using the same technique. A gastric pouch was fashioned over a 36Fr bougie, 5–7 cm long with a combined Biliopancreatic (BP) and alimentary limb of 150 cm (either 75 cm and 75 cm or 100 cm BP and 50 cm alimentary). SAGB was performed by two surgeons at one facility using the same technique. A gastric pouch was fashioned over a 36Fr bougie, commencing at the antrum 2 cm below the crow’s feet (Angular incisure). The BP was 150 cm from duodenojejunal flexure.

### Model of care for micronutrient management, including clinical and biochemical assessments

All participants received a consistent micronutrient management model of care. Details of this model of care are in Table 1. To note, nutritional biochemical assessment was carried out according to usual clinical practice and was consistent across the three services. This includes a standardised minimum micronutrient panel for each surgical procedure, determined on assessment of clinical guideline recommendations, including strength of the evidence and MDT consensus of risk within the clinical context of procedure type, limb length and population characteristics [2, 3]. Additionally, C-reactive protein (CRP) was collected as a measure of inflammation. The degree of inflammation has been shown to confound the validity of serum micronutrient tests differently, therefore, inflammation was defined by CRP cut offs specific to each micronutrient: >20 mg/L for zinc, >10 mg/L for vitamin A and vitamin B1 and C, >5 mg for all other [9, 15, 16]. HbA1c (%) was collected as a measure of glycaemic control. These biochemical results were gathered from three different pathology facilities available to the participants in the community. Details of micronutrient test assays, coefficient of variation (CV) and definitions for deficiency for all micronutrients screened are found in Table S2. De novo deficiency was defined as a new deficiency that developed since the last biochemical micronutrient blood test. Average weekly supplement intakes were compared to international guideline supplementation target guides (Table S3) [2, 3].

**Table 1:** Micronutrient management model of care, including clinical and biochemical assessments.

Micronutrient testing frequency	Preoperative, 1–3, 6, and 12 months postoperatively
Standardised minimum micronutrient panel	Sleeve gastrectomy: ferritin, transferrin saturation, vitamin B12, folate, copper, ceruloplasmin, 25-OH vitamin D, vitamin B1 and tests relating to nutrients of concern including haemoglobin, parathyroid hormone, albumin, C-Reactive Protein. Gastric bypass: all the above sleeve gastrectomy tests with additional vitamin A, retinol binding protein, vitamin E, vitamin C, zinc, plasma glutathione peroxidase (plgpx)^a^ and red blood cell glutathione peroxidase (rbcgpx)^a^. Any additional testing frequency or nutrients to be requested as per clinical judgement.
Multidisciplinary team appointment timeframes	Preoperative, 1–3, 6 and 12 months postoperatively, and includes dietetics and medical/surgical appointments.
Pre-operative nutrition assessments	The team dietitian provided pre-operative nutrition education on planned micronutrient management protocol, including monitoring schedule and prophylactic supplementation plans.
Micronutrient intake assessments	Carried out by the dietitian. Includes monitoring of micronutrient intake from both diet and supplementation. Dietary intake was globally assessed and quantification of intake (energy, protein, micronutrients at risk) estimated based on patients’ recall of food group frequencies across usual intake. Adequacy of intake was assessed by the dietitian using clinical judgement based on patient reported data [19–23]. Micronutrient supplement consumption (type, dose, frequency, including all oral, Intramuscular (IM) and Intravenous (IV) routes) was collected at each time point, including all prescribed and non-prescribed supplements. Usual weekly intake/exposure was divided by seven to calculate average daily intake of each micronutrient of interest. Average weekly intakes were compared to international guideline supplementation target guides (Table S3) [2, 3].
Pre-operative micronutrient supplementation protocol	Preoperative micronutrient supplementation prescription was individualised, targeted only to those with confirmed pre-operative deficient or insufficient results, and micronutrient correction not necessary for surgery to progress.
Post-operative micronutrient supplementation protocol	All participants were recommended prophylactic micronutrient supplementation of two standard multivitamins per day (Centrum Advance^TM^, GSK Australia), irrespective of serum micronutrient status. Nutrient doses provided by two Centrum AdvanceTM and comparison of these doses to perioperative guideline recommended target doses can be found in Table S1 (Supplementary materials) [2, 3]
	If serum deficiencies were identified postoperatively, additional supplementation was individualised, with no standardised protocol for supplementation embedded into usual care. Individual recommendations were based on an assessment of nutrition status and clinical judgement of usual dietary intakes and supplement use and micronutrient supplementation targets as defined by international guidelines for each nutrient [2, 3]. For example, additional vitamin B12 supplementation was individualised, depending on serum vitamin B12 levels and diet.

^a^Selenium status was measured using two functional markers, plasma glutathione peroxidase (plGPx) and red blood cell glutathione peroxidase (rbcGPx). A low plGPx level is thought to be reflective of short-term selenium deficiency and rbcGPx, longer term.

Anthropometric measures included weight (kg; calibrated scales), height (cm), body mass index (BMI; kg/m^2^), % Total Weight Loss (TWL) and % Excess Weight loss (EWL). Additional demographic data included sex (M/F), age (years), family history of obesity (Y/N), smoking history (Y/N), employment status (unemployed/retired/part time work/full time work/ student), socioeconomic status (quintiles) [17], hospital site, pre-operative obesity-related comorbidities and use of metformin (Y/N).

### Statistical analysis

All data were analysed using SPSS software (version 25 SPSS Inc., Chicago, IL. USA). Results were analysed by surgical type, including an initial separation of RYGB and SAGB participants. However, given no significant differences in micronutrient outcomes were found between RYGB and SAGB groups, results are presented together, as the GB cohort. Differences between SG and GB was carried out using the Mann-Whitney U test. Data imputation for missing values did not occur. Continuous variables that were normally distributed are presented as mean ± SD. Skewed data are presented as median (IQR). The paired *t*-test was used (after log transformation for skewed variables) to detect change over time. For normally distributed variables change is summarised, while for skewed data, symmetric percentage change is presented [18]. Changes over time for binary variables was carried out using McNemar’s test. Longitudinal multivariable analysis was carried out using Generalised Estimated Equations (GEE) for continuous (linear regression) and binary outcomes (logistic regression). Model 1 included time only (preoperative compared to 1–3 months, 6 months and 12 months) for all nutrients, while Model 2 included time and oral supplementation dose (log_e_(supplementation + 1)) and the presence of inflammation [9, 15, 16] for all nutrients with sufficient numbers to adequately model. Additional variables in Model 2 included sex for iron deficiency analysis, Intramuscular (IM) vitamin B12 supplementation (Y/N) and metformin use (Y/N) for vitamin B12 analysis and HbA1c (%) for vitamin D, plasma glutathione peroxidase (plGPx) and red blood cell glutathione peroxidase (rbcGPx) analysis [10–14]. Dietary micronutrient intake was not included as the available methodologies were deemed to be invalid in a pragmatic study in clinical practice [19–23]. Statistical significance was determined with *p* < 0.05.

## Results

### Demographics

**Fig. 1:**
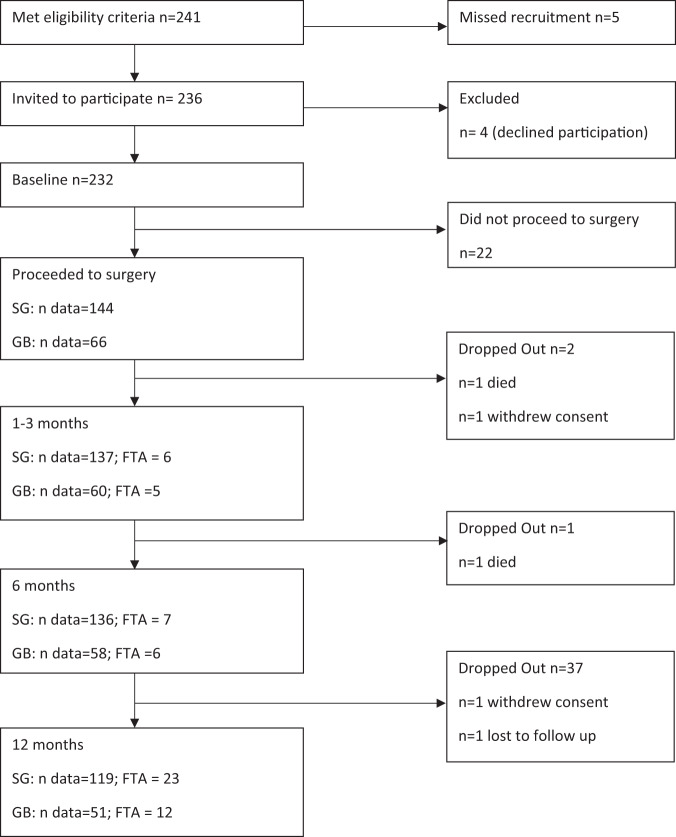
Participant recruitment and retention: Sleeve Gastrectomy and Gastric Bypass. SG sleeve gastrectomy; GB gastric bypass; n = number of participants enrolled or excluded at each stage of recruitment and follow-up data collection.

Participant recruitment and retention is shown in Fig. 1. A total 144 participants underwent a SG and 66 participants, a GB procedure. Overall study retention at 12 months was 81% (SG 84% and GB 76% respectively).

At baseline, participants receiving a SG had a mean age of 43 ± 10 years, 79% female, mean weight 140 ± 25 kg and mean BMI 50 ± 7 kg/m^2^ and 39% had pre-operative Type 2 Diabetes Mellitus (Table S4). Participants who underwent GB had a mean baseline age of 50 ± 10 years, 62% female, weight 138 ± 25 kg and BMI 49 ± 9 kg/m^2^ and 62% had pre-operative Type 2 Diabetes Mellitus (Table S4).

Anthropometric changes over time show significant sustained reductions in BMI, %TWL at 12 months (SG: 25%, GB: 27%) and %EWL at 12 months for both surgical procedures (Table S5). Additionally, glycaemic control improved at all time-points after surgery (Table S6).

### Oral micronutrient supplementation

**Fig. 2:**
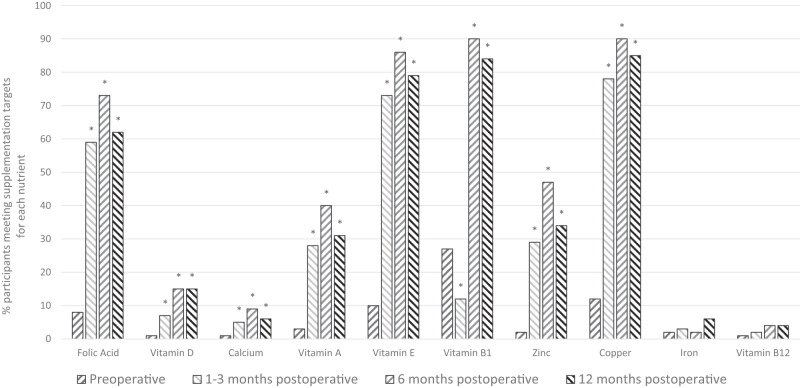
The proportion of participants having ‘adequate’ nutrient supplement intake. ^*^p < 0.05 change from preoperative intake, using the McNemar’s test.

The consumption of micronutrient supplements increased after surgery and was sustained at 12 months compared to pre-operative levels. (Table S7). The proportion of participants reaching supplement consumption targets at each time point is shown in Fig. 2. No nutrient reached published micronutrient supplementation targets (Table S3) for all participants at any time point. Additionally, there was large variation in multivitamin supplements consumed by participants that deviated from the recommended standard multivitamin supplementation. Across the cohort, 29 different supplement types were consumed preoperatively: 31 at 1–3 months; 33 at 6 months; and 26 at 12 months post-operatively. Individual nutrient content of the different supplement types varied, with consistently lower levels of vitamin A, selenium and copper, when compared to Centrum Advance^TM^ (Table S8). Descriptive analysis of micronutrient supplementation interventions to treat deficiencies can be found in supplementary materials.

### Inflammation

C-reactive protein decreased at all postoperative time points for both SG and GB (Table S6). The proportion of participants who had elevated CRP consistent with active inflammation (CRP > 5 mg/L), significantly reduced by 12 months (SG, 66% to 14%; *p* < 0.001 and GB, 70% to 14%; *p* < 0.001) (Table 2).

**Table 2:** Multivariable linear regression results for average nutritional biochemistry results and related independent variables for Sleeve Gastrectomy and Gastric Bypass participants.

Dependent Variable	Independent Variable^a^	Sleeve Gastrectomy		Gastric Bypass	
		Difference (95% CI)^b^	*p* value	Difference (95% CI)^b^	*p* value
Ferritin (ug/L)^f^	Preop	ref		ref	
	1–3 months postop	16% (−1%, 33%)	0.07	21% (0%, 43%)	0.05
	6 months postop	−3% (−20%, 13%)	0.7	−10% (−38%, 18%)	0.5
	12 months postop	−12% (−32%, 8%)	0.2	−32% (−65%, 1%)	0.06
	Oral iron supplement dose^c^	−1% (−8%, 6%)	0.8	10% (1%, 20%)	0.02
	Female	−47% (−82%, −12%)	0.008	−77% (−117%, −36%)	<0.001
	CRP > 5 mg/L	15% (0%, 30%)	0.05	32% (9%, 54%)	0.006
Transferrin Saturation (%)^f^	Preop	ref		ref	
	1–3 months postop	4.0 (2.0, 6.0)	<0.001	2.4 (0.0, 4.7)	0.05
	6 months postop	6.4 (3.8, 9.0)	<0.001	4.8 (1.7, 7.9)	0.002
	12 months postop	6.5 (3.8, 9.3)	<0.001	6.8 (3.4, 10.2)	<0.001
	Oral iron supplement dose^c^	−0.1 (−0.9, 0.7)	0.8	−0.2 (−1.4, 0.9)	0.7
	Female	−2.2 (−6.1, 1.6)	0.3	0.1 (−2.5, 2.7)	0.9
	CRP > 5 mg/L	−3.5 (−6.0, −1.1)	0.004	−2.0 (−4.3, 0.3)	0.08
Vitamin B12 (pmol/L)	Preop	ref		ref	
	1–3 months postop	−2% (−14%, 10%)	0.8	13% (−1%, 27%)	0.08
	6 months postop	−8% (−21%, 4%)	0.2	−7% (−26%, 12%)	0.5
	12 months postop	−5% (−20%, 10%)	0.5	−11% (−31%, 9%)	0.3
	Oral vitamin B12 supplement dose^c^	4% (0%, 8%)	0.03	4% (0%, 8%)	0.04
	IM vitamin B12 supplementation^d^	92% (47%, 138%)	<0.001	102% (69%, 135%)	<0.001
	Metformin^e^	2% (−10%, 14%)	0.7	1% (−13%, 15%)	0.9
	CRP > 5 mg/L	6% (−4%, 16%)	0.3	10% (−4%, 23%)	0.2
Folate (nmol/L)	Preop	ref		ref	
	1–3 months postop	−2.7 (−5.9, 0.5)	0.1	−5.4 (−10.0, −0.7)	0.02
	6 months postop	−3.4 (−7.3, 0.5)	0.09	−3.7 (−9.1, 1.8)	0.2
	12 months postop	−4.1 (−8.2, 0.1)	0.06	−4.1 (−10.4, 2.1)	0.2
	Oral folic acid supplement dose^c^	1.9 (1.4, 2.5)	<0.001	1.4 (0.7, 2.2)	<0.001
	CRP > 5 mg/L	−0.3 (−3.2, 2.5)	0.8	−3.4 (−7.4, 0.7)	0.1
Copper (umol/L)	Preop	ref		ref	
	1–3 months postop	−4.0% (−8.4%, 0.4%)	0.07	−1.2% (−6.1%, 3.7%)	0.6
	6 months postop	−1.0% (−6.0%, 3.9%)	0.7	−3.2% (−9.5%, 3.0%)	0.3
	12 months postop	−2.3% (−7.2%, 2.7%)	0.4	−7.2% (−13.6%, −0.9%)	0.02
	Oral copper supplement dose^c^	0.3% (−0.5%, 1.0%)	0.4	0.1% (−0.7%, 0.8%)	0.8
	CRP > 5 mg/L	11.3% (7.2%, 15.3%)	<0.001	8.5% (4.7%, 12.4%)	<0.001
Ceruloplasmin (umol/L)	Preop	ref		ref	
	1–3 months postop	−4% (−9%, 1%)	0.2	0% (−4%, 4%)	1
	6 months postop	−2% (−8%, 4%)	0.5	−3% (−9%, 2%)	0.3
	12 months postop	−2% (−9%, 4%)	0.5	−9% (−15%, −3%)	0.002
	Oral copper supplement dose^c^	−0.13% (−1.02%, 0.76%)	0.8	0.00% (−0.61%, 0.61%)	1
	CRP > 5 mg/L	11% (6%, 16%)	<0.001	10% (6%, 14%)	<0.001
Vitamin D (nmol/L)	Preop	ref		ref	
	1–3 months postop	13.9 (6.8, 20.9)	<0.001	−6.7 (−13.4, −0.1)	0.05
	6 months postop	10.4 (2.9, 19.9)	0.007	−7.4 (−16.7, 1.9)	0.1
	12 months postop	10.5 (1.0, 19.9)	0.03	−5.9 (−17.6, 5.8)	0.3
	Oral vitamin D supplement dose^c^	0.5 (−0.5, 1.5)	0.3	3.0 (1.9, 4.2)	<0.001
	CRP > 5 mg/L	2.8 (−2.1, 7.7)	0.3	6.7 (−3.4, 16.6)	0.2
	HbA1c (%)	1.3 (−1.5, 4.1)	0.4	−0.6 (−2.9, 1.7)	0.6
Vitamin B1 Hb (nmol/g Hb)	Preop	ref		ref	
	1–3 months postop	−11% (−20%, −1%)	0.03	−24% (−31%, −16%)	<0.001
	6 months postop	−3% (−13%, 8%)	0.6	−9% (−18%, −1%)	0.05
	12 months postop	2% (−8%, 12%)	0.7	−6% (−15%, 2%)	0.1
	Oral vitamin B1 supplement dose^c^	7% (2%, 13%)	0.007	11% (8%, 14%)	<0.001
	CRP > 10 mg/L	10% (2%, 17%)	0.02	4% (−4%, 11%)	0.4
Vitamin A (umol/L)	Preop			ref	
	1–3 months postop			−0.37 (−0.50, −0.23)	<0.001
	6 months postop			−0.28 (−0.42, −0.13)	<0.001
	12 months postop			−0.18 (−0.35, −0.01)	0.04
	Oral vitamin A supplement dose^c^			−0.014 (−0.031, 0.002)	0.1
	CRP > 10 mg/L			−0.13 (−0.24, −0.02)	0.02
Retinol Binding Protein (umol/L)	Preop			ref	
	1–3 months postop			−0.50 (−0.64, −0.29)	<0.001
	6 months postop			−0.33 (−0.51, −0.29)	<0.001
	12 months postop			−0.2 (−0.4, 0.0)	0.06
	Oral vitamin A supplement dose^c^			−0.021 (−0.041, −0.001)	0.04
	CRP > 5 mg/L			−0.02 (−0.18, 0.13)	0.8
Vitamin E (umol/L)	Preop			ref	
	1–3 months postop			−10.7 (−14.9, −6.6)	<0.001
	6 months postop			−6.3 (−10.7, −1.9)	0.005
	12 months postop			−6.1 (−10.1, −2.2)	0.002
	Oral vitamin E supplement dose^c^			0.86 (−0.03, 1.74)	0.06
	CRP > 5 mg/L			3.46 (−0.07, 6.97)	0.06
Vitamin C (umol/L)	Preop			ref	
	1–3 months postop			−2.9 (−12.1, 6.4)	0.5
	6 months postop			2.3 (−8.3, 12.9)	0.7
	12 months postop			5.1 (−6.5, 16.6)	0.4
	Oral vitamin C supplement dose^c^			2.7 (0.7, 4.7)	0.008
	CRP > 10 mg/L			−2.4 (−10.5, 5.6)	0.6
Zinc (umol/L)	Preop			ref	
	1–3 months postop			0.67 (−0.03, 1.37)	0.06
	6 months postop			−1.44 (−2.43, −0.45)	0.005
	12 months postop			−1.10 (−1.82, −0.38)	0.003
	Oral zinc supplement dose^c^			0.29 (0.06, 0.52)	0.02
	CRP > 20 mg/L			−1.08 (−2.54, 0.39)	0.2
Plasma Glutathione Peroxidase (U/L)	Preop			ref	
	1–3 months postop			−6% (−17%, 4%)	0.3
	6 months postop			−5% (−18%, 7%)	0.4
	12 months postop			14% (2%, 26%)	0.03
	Oral selenium supplement dose^c^			−0.3% (−2.6%, 2.0%)	0.8
	CRP > 5 mg/L			−9% (−17%, −1%)	0.03
	HbA1c (%)			7% (5%, 9%)	<0.001
Red Cell Glutathione Peroxidase (u/g Hb)	Preop			ref	
	1–3 months postop			3% (−5%, 11%)	0.5
	6 months postop			−1% (−12%, 10%)	0.9
	12 months postop			−5% (−16%, 5%)	0.3
	Oral selenium supplement dose^c^			0.4% (−1.6%, 2.4%)	0.7
	CRP > 5 mg/L			−1% (−9%, 6%)	0.7
	HbA1c (%)			1% (−2%, 3%)	0.6

*ref* reference category.

^a^Using the Generalised Estimated Equation.

^b^Difference or % difference, where % difference was used for nutrition biochemistry variables that were logged due to not being normally distributed.

^c^Oral supplementation dose is log_e_(supplementation dose/day +1) as is not normally distributed and contains zero values.

^d^Patient received any dose of IM vitamin B12 supplementation prior to their blood test at that timepoint.

^e^On any dose of metformin at time of blood test.

^f^Cases that used IV iron excluded from analysis.

### The impact of supplementation and inflammation on biochemical micronutrient markers (Table 2)

#### Micronutrient supplementation

Multivariable analysis found supplementation to be protective for vitamin B1 after both procedures. Supplementation maintained vitamin B12 levels, with serum vitamin B12 positively associated with oral and IM routes of administration. After SG, no other serum nutrient level was found to be independently affected by supplementation dose. After GB, supplementation dose was independently positively associated with serum levels of ferritin, folate, vitamin D, vitamin C and zinc. However, no effect was found for transferrin saturation, copper, vitamin E, plGPx and rbcGPx levels. Vitamin A supplementation was associated with higher retinol binding protein, but not vitamin A levels.

#### Inflammation

There was an independent relationship between inflammation and the biochemical markers of some micronutrients. In the setting of inflammation, serum copper increased by an average of 11% [95% CI 7, 15%] (*p* < 0.001), after SG, and by an average of 9% [95% CI 5, 12%] (*p* = 0.01) after GB, with ceruloplasmin mirroring these results after both procedures. Ferritin appeared to increase by an average of 15% [95%CI 0,30%)] (*p* = 0.05) after SG and by an average of 32% [95%CI 9, 54%] (*p* = 0.006) after GB. Vitamin B1 increased by an average 10% [95% CI 2, 17%] (*p* = 0.02) in the setting of inflammation, after SG. This relationship was not observed after GB. Vitamin A, only tested after GB, was lower by an average of 0.13 umol/L [95% CI 0.02, 0.24 umol/L] (*p* = 0.02), in the setting of inflammation. PlGPx, also only tested after GB, may be lower in the setting of inflammation.

### Micronutrient deficiency outcomes

#### Univariable associations between surgery and nutrient deficiency

After SG, no nutrients had an increase in deficiency prevalence in the first postoperative year, and rates of vitamin D deficiency decreased at all post-operative timepoints, compared to pre-operative deficiency rates. However, after GB, there was an increase in vitamin B1 and vitamin A deficiency at 1–3 months after surgery (Table 3). Additionally, prevalence of low plGPx increased in this same period. Conversely, rates of vitamin B12 insufficiency decreased at 1–3 and 6 months after GB, when compared to pre-operative prevalence.

**Table 3:** Prevalence of nutrient deficiency from preoperative up to 12 months after Sleeve Gastrectomy and Gastric Bypass.

Nutritional biochemistry test	Cut off for deficiency	Sleeve Gastrectomy				Gastric Bypass			
		Preoperative (*n* = 90–126)	1–3 months postoperative (*n* = 92–130)	6 months postoperative (*n* = 94–130)	12 months postoperative (*n* = 86–118)	Preoperative (*n* = 63–66)	1–3 months postoperative (*n* = 53–64)	6 months postoperative (*n* = 52–56)	12 months postoperative (*n* = 42–48)
Haemoglobin	M: <130 g/L F: <120 g/L	10 (8%)	12 (9%)	10 (8%)	11 (9%)	14 (21%)	11 (17%)	13 (23%)	3 (7%)*
Ferritin	<30 ug/L	16 (13%)	17 (14%)	22 (19%)	23 (22%)	11 (17%)	7 (12%)	9 (17%)	13 (27%)
Transferrin Saturation	<16%	56 (44%)	26 (21%)***	24 (20%)***	21 (20%)**	34 (52%)	23 (39%)*	15 (28%)**	6 (13%)***
Iron deficiency	Ferritin <30 ug/L & Trans sat <16%	9 (7%)	8 (7%)	11 (10%)	13 (12%)	8 (12%)	8 (12%)	6 (9%)	4 (6%)
Vitamin B12	<110 pmol/L^a^	0 (0%)	3 (3%)	4 (3%)	3 (3%)	2 (3%)	0 (0%)	1 (2%)	2 (4%)
	110–133 pmol/L^b^	4 (3%)	4 (3%)	5 (4%)	6 (6%)	13 (20%)	1 (2%)**	4 (7%)*	6 (13%)
Folate	<10 nmol/L	3 (3%)	3 (2%)	11 (9%)	8 (8%)	2 (3%)	1 (2%)	5 (9%)	3 (6%)
Copper	<11 umol/L	0 (0%)	0 (0%)	1 (1%)	0 (0%)	0 (0%)	0 (0%)	0 (0%)	0 (0%)
Ceruloplasmin	<1.48 umol/L	0 (0%)	4 (4%)	2 (2%)	2 (3%)	1 (2%)	0 (0%)	0 (0%)	1 (2%)
Vitamin D	<30 nmol/L^c^	3 (2%)	1 (1%)	0 (0%)	2 (2%)	4 (6%)	1 (2%)	0 (0%)	2 (4%)
	30–50 nmol/L^d^	35 (27%)	7 (6%)***	8 (7%)***	10 (9%)***	16 (24%)	10 (17%)	8 (15%)	10 (21%)
Parathyroid hormone	>7 pmol/L	29 (25%)	17 (14%)	14 (12%)	13 (14%)	11 (17%)	12 (20%)	9 (17%)	14 (29%)
Vitamin B1	<0.9 nmol/g Hb	8 (9%)	13 (13%)	10 (9%)	1 (1%)	4 (6%)	12 (21%)**	6 (11%)	2 (4%)
Vitamin A	<1.1 umol/L					2 (3%)	11 (21)**	7 (13%)	2 (4%)
Retinol Binding Protein	<1.4 umol/L					3 (5%)	12 (21%)**	10 (19%)*	4 (9%)
Vitamin E	<11 umol/L					0 (0%)	0 (0%)	0 (0%)	0 (0%)
Vitamin C	<11 umol/L					2 (3%)	2 (5%)	2 (4%)	0 (0%)
Zinc	<8 umol/L					0 (0%)	0 (0%)	2 (4%)	1 (2%)
plGPx	<370 U/L					25 (39%)	33 (59%)*	28 (53%)	11 (23%)
rbcGPx	<25 U/g Hb					5 (8%)	4 (7%)	4 (8%)	6 (13%)

*trans sat* transferrin saturation, *plGPx* plasma glutathione peroxidase, *rbcGPx* red blood cell glutathione peroxidase.

*p < 0.05; **p ≤ 0.01, ***p < 0.001 using McNemar’s test.

^a^Vitamin B12 deficiency.

^b^Vitamin B12 insufficiency.

^c^Vitamin D deficiency.

^d^Vitamin D insufficiency.

#### De novo deficiency

After SG, de novo deficiency developed at a rate of 0–5% for each nutrient at each timepoint. After GB, up to 25% of deficiency cases were deemed de novo deficiency across all time points, most occurring early, at 1–3 months after surgery. This occurred for selenium (as measured by plGPx) (25%), vitamin D (10%), vitamin B1 (16%) and vitamin A (13%); however, appeared temporary for all but de novo selenium deficiencies (in whom 4% maintained a reduced plGPx at 12 months). De novo low ferritin levels were not detected at 1–3 months however were seen in 6% of patients by 12 months. No de novo deficiency was identified for copper or vitamin E. No statistical analysis was possible for de novo deficiencies, due to low numbers.

### Independent relationship between surgery and nutrient deficiency: multivariable analysis

#### Sleeve gastrectomy

Once the role of supplementation and inflammation was accounted for, there was an increased risk of folate deficiency at 6 months, and a potential for iron deficiency 12 months after surgery (Table 4). However, rates of vitamin B12, copper and thiamine deficiency were not impacted by surgery. Vitamin D deficiency risk decreased at all timepoints after surgery.

**Table 4:** Multivariable logistic regression results (using GEE) for individual nutrient deficiency and related independent variables for Sleeve Gastrectomy and Gastric Bypass participants.

Dependent Variable	Independent Variable^a^	Sleeve Gastrectomy				Gastric Bypass			
		Model 1^e^		Model 2^f^		Model 1^e^		Model 2^f^	
		Odds ratio (95% CI)	*p* value	Odds ratio (95% CI)	*p* value	Odds ratio (95% CI)	*p* value	Odds ratio (95% CI)	*p* value
Iron deficiency (Ferritin <30 ug/L and Transferrin Saturation <16%)^g^	Preop	Ref		Ref		Ref		Ref	
	1–3 months postop	1.30 (0.64, 2.64)	0.5	1.49 (0.38, 5.87)	0.6	0.73 (0.44, 1.23)	0.2	0.82 (0.32, 2.11)	0.7
	6 months postop	1.47 (0.71, 3.07)	0.3	2.05 (0.59, 7.06)	0.3	0.79 (0.39, 1.57)	0.5	1.28 (0.35, 4.70)	0.7
	12 months postop	1.66 (0.81, 3.40)	0.2	4.11 (1.04, 16.2)	0.04	0.83 (0.36, 1.88)	0.7	1.17 (0.35, 3.97)	0.8
	Oral iron supplement dose^b^			1.02 (0.74, 1.40)	0.9			0.75 (0.47, 1.21)	0.2
	Female			3.52 (0.45, 27.4)	0.2			5.37 (1.06, 27.1)	0.04
	CRP >5mg/L			1.90 (1.04, 3.44)	0.04			0.80 (0.32, 2.03)	0.6
Vitamin B12 deficiency and insufficiency (<133 pmol/L)	Preop	Ref		Ref		Ref			
	1–3 months postop	1.08 (0.25, 4.57)	0.9	0.73 (0.11, 4.69)	0.7	0.14 (0.03, 0.62)	0.01		
	6 months postop	1.75 (0.58, 5.29)	0.3	1.24 (0.26, 5.89)	0.8	0.30 (0.10, 0.94)	0.04		
	12 months postop	2.07 (0.58, 7.42)	0.3	2.39 (0.46, 12.3)	0.3	0.54 (0.20, 1.44)	0.2		
	Oral vitamin B12 supplement dose^b^			1.05 (0.69, 1.60)	0.8				
	IM vitamin B12 supplementation^c^			1.16 (0.10, 13.9)	0.9				
	Metformin^d^			2.78 (0.94, 8.27)	0.07				
	CRP >5mg/L			1.29 (0.48, 3.43)	0.6				
Folate deficiency (<10 nmol/L)	Preop	Ref		Ref		Ref		Ref	
	1–3 months postop	0.95 (0.20, 4.65)	1	2.91 (0.39, 21.7)	0.3	0.65 (0.08, 5.45)	0.7	0.76 (0.07, 7.94)	0.8
	6 months postop	3.80 (1.14, 12.7)	0.03	12.9 (2.00, 83.7)	0.007	3.30 (1.07, 10.2)	0.04	8.56 (1.64, 44.6)	0.01
	12 months postop	1.09 (0.89, 10.7)	0.08	3.68 (0.64, 21.9)	0.1	2.00 (0.28, 14.6)	0.5	7.67 (1.10, 53.7)	0.04
	Oral folic acid supplement dose^b^			0.78 (0.62, 0.98)	0.04			0.93 (0.74, 1.17)	0.5
	CRP >5mg/L			1.22 (0.37, 4.00)	0.7			9.46 (2.20, 40.7)	0.003
Vitamin D deficiency and insufficiency (<50 nmol/L)	Preop	Ref		Ref		Ref		Ref	
	1–3 months postop	0.15 (0.06, 0.37)	<0.001	0.21 (0.07, 0.66)	0.008	0.62 (0.27, 1.41)	0.25	1.93 (0.64, 5.82)	0.2
	6 months postop	0.20 (0.09, 0.41)	<0.001	0.16 (0.05, 0.56)	0.004	0.57 (0.24, 1.36)	0.21	3.19 (0.88, 11.5)	0.08
	12 months postop	0.27 (0.14, 0.54)	<0.001	0.21 (0.05, 0.82)	0.02	0.80 (0.33, 1.94)	0.63	5.10 (1.24, 20.9)	0.03
	Oral vitamin D supplement dose^b^			1.03 (0.88, 1.21)	0.7			0.74 (0.65, 0.85)	<0.001
	CRP >5mg/L			1.32 (0.60, 3.00)	0.5			1.83 (0.75, 4.49)	0.2
	HbA1c (%)			0.91 (0.68, 1.22)	0.5			0.96 (0.69, 1.33)	0.8
Vitamin B1 deficiency (<0.9 nmol/g Hb)	Preop	Ref		Ref		Ref		Ref	
	1–3 months postop	0.64 (0.27, 1.48)	0.3	1.87 (0.69, 5.07)	0.2	0.25 (0.10, 0.63)	0.003	8.27 (2.67, 25.6)	<0.001
	6 months postop	1.00 (0.41, 2.41)	1	1.52 (0.42, 5.59)	0.5	0.52 (0.18, 1.49)	0.2	6.27 (1.86, 21.1)	0.003
	12 months postop	6.60 (1.18, 36.9)	0.03	0.24 (0.05, 1.22)	0.09	1.54 (0.30, 8.27)	0.6	2.04 (0.30, 13.9)	0.5
	Oral vitamin B1 supplement dose^b^			0.49 (0.27, 0.88)	0.02			0.45 (0.29, 0.70)	<0.001
	CRP >10mg/L			0.17 (0.05, 0.59)	0.005			1.78 (0.73, 4.36)	0.2
Vitamin A deficiency (<1.1 umol/L)	Preop					Ref		Ref	
	1–3 months postop					6.31 (2.02, 19.8)	0.002	5.25 (0.93, 29.5)	0.06
	6 months postop					3.56 (1.24, 10.2)	0.02	2.99 (0.53, 16.8)	0.2
	12 months postop					0.78 (0.12, 5.02)	0.8	0.79 (0.10, 6.43)	0.8
	Oral vitamin A supplement dose^b^							1.11 (0.91, 1.37)	0.3
	CRP >10mg/L							3.25 (1.04, 10.2)	0.04
Plasma Glutathione Peroxidase deficiency (<370 U/L)	Preop					Ref		Ref	
	1–3 months postop					2.38 (1.35, 4.18)	0.003	1.74 (0.64, 4.79)	0.3
	6 months postop					1.83 (1.03, 3.24)	0.04	1.43 (0.40, 5.08)	0.6
	12 months postop					0.54 (0.28, 1.03)	0.06	0.38 (0.11, 1.32)	0.1
	Oral selenium supplement dose^b^							0.92 (0.75, 1.13)	0.4
	CRP >5mg/L							2.27 (1.16, 4.44)	0.02
	Hba1c (%)							0.51 (0.37, 0.71)	<0.001
Red Cell Glutathione Peroxidase deficiency (<25 U/g Hb)	Preop					Ref		Ref	
	1–3 months postop					1.01 (0.50, 2.01)	1	1.09 (0.25, 4.66)	0.9
	6 months postop					1.14 (0.43, 3.04)	0.8	1.13 (0.15, 8.80)	0.9
	12 months postop					2.07 (0.79, 5.38)	0.1	2.27 (0.30, 17.3)	0.4
	Oral selenium supplement dose^b^							1.14 (0.85, 1.53)	0.4
	CRP >5mg/L							1.73 (0.33, 9.14)	0.5
	HbA1c (%)							1.20 (0.89, 1.64)	0.2

*ref* reference category.

^a^Using the Generalised Estimated Equation, comparing the indpendent variables to preoperative nutrient deficiency prevalence as the reference category.

^b^Oral supplementation dose is log_e_(supplementation dose/day +1) as is not normally distributed and contains zero values.

^c^Patient received any dose of IM vitamin B12 supplementation prior to their blood test at that timepoint.

^d^On any dose of metformin at time of blood test.

^e^Model 1: multivariable model with only time as the independent variable.

^f^Model 2: multivariable model with time, supplementation, inflammation (measured using C-reactive protein (CRP) cut offs to define inflammation) and metformin for vitamin B12 as independent variables.

^g^Cases that used IV iron excluded from analysis.

#### Gastric bypass

Once any potential role of supplementation and inflammation was accounted for, the risk of vitamin B1 deficiency appeared to increase at both 1–3 and 6 months after surgery and the risk of folate deficiency appeared to increase 6 months after surgery. There was a potential increased risk of folate and vitamin D deficiency 12 months after surgery. Low plGPx was independently related to improvements in glycaemic control after surgery rather than time since surgery. However, GB was not associated with an increased risk of iron, copper, vitamin E, vitamin C and zinc deficiency or low long term selenium stores (rbcGPx). There were insufficient numbers of vitamin B12 deficiency to analyse.

## Discussion

In the setting of prophylactic post-surgical micronutrient prescription, this study found the majority of micronutrients did not appear vulnerable to increased deficiency prevalence in the first 12 months after bariatric surgery, even when supplement targets were, mostly, not met. The low rates of surgical-induced micronutrient deficiency were observed within a model of care where participants received frequent and consistent expert multidisciplinary care pre- and post-operatively, including education on prophylactic supplementation, and monitoring of biochemical micronutrient markers, dietary intake and postoperative surgical complications.

These results indicate that de novo deficiency was uncommon, with higher risk for deficiencies identified only in some key nutrients. Although the need for prophylactic micronutrient supplementation continues, these results may begin to challenge the need for high doses of all ten nutrients within prophylactic multivitamin prescription after SG or GB, where the limb length is ≤150 cm, particularly when patients are supported in a multidisciplinary post-operative setting [2, 3]. Although supplementation intake increases after surgery, doses of, vitamin B12 and iron were met by <20% of patients without an increasing incidence of deficiency. Additionally, vitamin D deficiency decreased after SG despite less than one third of participants meeting vitamin D supplement targets at any timepoint. However, these results indicate that supplementation may only have an independent role in preventing deficiency in folate (after both procedures) and vitamin B1 (after GB). This may suggest that prioritising high doses of these nutrients within a multivitamin is recommended. In addition, further investigation into the contribution of micronutrients from dietary intake for inclusion in multivariable analysis is recommended for future research. Overall, the lower than expected risk for micronutrient deficiencies despite lower than prescribed exposure to supplementation found in this study challenges current blanket recommendations for high dose broad prophylactic supplementation.

The independent relationship between inflammation and ferritin is recognised [24], however, the impact of chronic obesity-related inflammation and surgery on other micronutrients remains poorly recognised. Prevalence of inflammation was high pre-operatively and bariatric surgery had a remarkable dampening effect by 6–12 months [25]. This is the first study to consider the impact of inflammation when interpreting micronutrient status before and after bariatric surgery. In the presence of inflammation ferritin, copper and vitamin B1 levels appeared to increase while vitamin A and potentially plGPx levels decreased. While this association has been observed in the general population for ferritin, vitamin A, vitamin B1 and copper [9, 16, 24], little data exists in a bariatric surgical setting. Furthermore, plGPx’s independent relationships with glycaemic control and inflammation reflect its role in oxidative stress [11, 12]. Although selenium is a rate limiting factor to plGPx activity if body stores are low, this finding suggests that in a population with increased oxidative stress or high HbA1c, this functional marker may not be the most reliable measure of selenium status and should be interpreted with caution [11, 12]. Overall, this study highlights that an assessment of systemic inflammation is important when interpreting micronutrient status in a bariatric population, which is not included in current perioperative bariatric surgery guidelines [2, 3].

These results may also inform rationalising the testing frequency of some nutrients in the first postoperative year. American Society for Metabolic and Bariatric Surgery practice guidelines recommend testing up to 10 nutrients, up to four times within the first post-operative year [2]. However, more recent British Obesity and Metabolic Surgery Society guidelines, have moved towards a more individualised testing schedule, and suggest only five nutrients up to three times in the first year, with other nutrients be tested as required or annually [3]. This study found that after SG, only folate and potentially iron, appeared vulnerable to deficiency 6 months after surgery. Additionally, after GB only vitamin B1 and folate appeared vulnerable to deficiency due to surgery, with uncertainty remaining about the potential risk vitamin D deficiency. While low plGPx and vitamin A deficiency prevalence did increase after surgery, both were independently related to inflammation and/or glycaemic control, not time since surgery, suggesting mechanisms other than surgically induced malabsorption are playing a role. Therefore, this study supports the notion of a more individualised approach to micronutrient monitoring in the first 12 months after surgery, in the context of an uncomplicated post-operative course, the absence of pre-operative micronutrient deficiencies, and care including regular dietitian involvement. This approach may reduce testing costs and burden on the individual and health service, while also maintaining safety [6]. Further research is recommended to investigate this further, in the short and long term.

It is acknowledged that access to multidisciplinary post-surgical support may be heterogeneous in other hospital settings [26, 27] and therefore the results of this study may not be generalisable to all surgical units. While the biochemical analysis of serum micronutrient levels were performed by more than one laboratory, and at times by more than one type of assay, this is reflective of real world practice. Clinical interpretation of results took a conservative approach with consideration of the width and lower limit of the 95% confidence intervals to determine whether the risk of micronutrient deficiency is great enough to support broad protocol driven postoperative monitoring of individual nutrients [28]. Furthermore, although detailed information was collected on supplementation dose, timing of dose in relation to the biochemical micronutrient test was not recorded and may confound some nutrient results. Additionally, dietary intake was not collected to the detail required to accurately assess micronutrient intake, thus supplement intake does not reflect total overall intake of each nutrient but rather that consumed via supplements only. In the context of vastly reduced oral intake after surgery, an investigation of food contribution to micronutrients in combination with supplements is warranted for future research

In conclusion, this study highlights novel relationships between bariatric surgery, micronutrient deficiencies, supplementation dose, and the presence of inflammation. Although surgery did increase the risk of micronutrient deficiency for some key nutrients in the first postoperative year, when accounting for other influencers, this is not the case for all. Therefore, this study begins to challenge the need for blanket high dose supplementation doses and testing frequencies for the 10 guideline-identified at risk nutrients in the first year after surgery. A more individualised, person-centred approach may be safe and efficacious, within a supportive MDT with expertise in bariatric surgery. However, standardised blanket protocols may still be needed in other clinical settings. Future research is recommended, to investigate this concept further.

### Supplementary information

Supplementary materials PDF

Supplementary table 1: Micronutrient content of Centrum Advanced

Supplementary table 2: Nutritional biochemistry test units of measure, definition of deficiency and testing methodologies of the three pathology laboratories included in this study; including assays

Supplementary table 6A: Nutritional biochemistry over time: Sleeve Gastrectomy

Supplementary table 6B: Nutritional biochemistry over time: Gastric Bypass

Supplementary table 7A: Average consumption of nutrients from oral supplementation over time: Sleeve Gastrectomy

Supplementary table 7B: Average consumption of micronutrients from oral supplementation over time after Gastric Bypass

Supplementary table8: Different multivitamin supplements consumed by participants during the study

## Data Availability

Additional raw data are available from the corresponding author on reasonable request.
