# Supplementary material for: Monitoring for micronutrient deficiency after bariatric surgery—what is the risk?
Source: Eur J Clin Nutr. 2023 Aug 7;77(11):1071–83. doi: 10.1038/s41430-023-01318-3 (PMC10630125; doi:10.1038/s41430-023-01318-3)
Supplement: Supplementary file 1 — Supplementary materials PDF [file 41430_2023_1318_MOESM1_ESM.pdf]

S3. International guideline recommended nutrient intake from supplementation after surgery

Nutrient	Sleeve Gastrectomy	Gastric Bypass
Elemental iron[1]	65mg	65mg
Vitamin B12[1]	Intra Muscular (IM)	Intra Muscular (IM)
Folic acid[1]	400ug M[a]<br>800ug F[b]	400ug M[a]<br>800ug F[b]
Vitamin D[1]	2,000IU	2,000IU
Calcium[2]	1,200mg[c]	1,200mg[c]
Vitamin A[1]	2,000IU	2,000IU
Vitamin E[2]	15mg	15mg
Vitamin C	no dose recommended	no dose recommended
Vitamin B1[1]	4.85mg for first 3-4mths<br>>4mths postop:<br>0.8mg Females<br>1mg Males	4.85mg for first 3-4mths<br>>4mths postop:<br>0.8mg Females<br>1mg Males
Zinc[1]	15mg	15mg
Copper[1]	2mg	2mg
Selenium	no dose recommended	no dose recommended

[1]O'Kane M, Parretti HM, Pinkney J, Welbourn R, Hughes CA, Mok J, et al. British Obesity and Metabolic Surgery Society Guidelines on perioperative and postoperative biochemical monitoring and micronutrient replacement for patients undergoing bariatric surgery—2020 update. Obes Rev. 2020;21(11):e13087-n/a. doi: 10.1111/obr.13087; [2]Mechanick JI, Apovian C, Brethauer S, Garvey WT, Joffe AM, Kim J, et al. Clinical practice guidelines for the perioperative nutrition, metabolic, and nonsurgical support of patients undergoing bariatric procedures–2019 update: cosponsored by American Association of Clinical Endocrinologists/American College of Endocrinology, The Obesity Society, American Society for Metabolic & Bariatric Surgery, Obesity Medicine Association, and American Society of Anesthesiologists. Surgery for Obesity and Related Diseases. 2020;16(2):175-247, [a]M: male; [b]F: female; [c]calcium recommendations of 1200mg are from supplements and diet

S4. Baseline demographics of study cohort

Baseline demographic		Sleeve Gastrectomy (n=144)	Gastric Bypass (n=66)	p value[a]
Female (n (%))		113 (79%)	41 (62%)	0.013
Age (years) (Mean [SD])		43 [10]	50 [10]	NS
Weight (kg) (Mean [SD])		140.0 [25.3]	137.8 [25.5]	NS
BMI (kg/m^2) (Mean [SD])		49.7 [7.4]	48.7 [8.8]	NS
Baseline comorbidities	Cardiovascular disease (n (%))	11 (8%)	6 (9%)	NS
	Hypertension (n (%))	71 (49%)	41 (62%)	NS
	Hyperlipidaemia (n (%))	50 (35%)	39 (59%)	0.001
	Gastroesophageal reflux disease (n (%))	46 (32%)	25 (38%)	NS
	Type 2 Diabetes Mellitus (n (%))	56 (39%)	44 (67%)	<0.001
	Obstructive Sleep Apnoea (n (%))	82 (57%)	36 (55%)	NS
Family History of Obesity (n (%))		107 (86%)	44 (69%)	0.006
History of smoking (n (%))		25 (17%)	31 (48%)	<0.001
Employment status	Unemployed/disability pension (n (%))	69 (48%)	42 (64%)	NS
	Retired (n (%))	4 (3%)	2 (3%)	
	Part time work (n (%))	24 (17%)	6 (9%)	
	Full time work (n (%))	38 (26%)	16 (24%)	
	Student (n (%))	8 (6%)	0 (0%)	
Socioeconomic status postcode percentiles	0-20% (n (%)) (most disadvantaged)	42 (29%)	15 (23%)	NS
	21-40% (n (%))	24 (17%)	15 (23%)	
	41-60% (n (%))	42 (60%)	27 (41%)	
	61-80% (n (%))	18 (13%)	9 (14%)	
	81-100% (n (%)) (most advantaged)	0 (0%)	0 (0%)	
Location of surgery	Site 1 (n (%))	43 (30%)	66 (100%)	<0.001
	Site 2(n (%))	101 (70%)	0 (0%)	

[a]Using the Mann-Whitney U test for continuous variables and chi-squared test for categorical variables.

S5 Anthropometry of study cohort over time

	Preoperative	1-3 months postoperative			6 months postoperative			12 months postoperative		
			Change from preoperative			Change from preoperative			Change from preoperative	
Anthropometry	Mean [SD]	Mean [SD]	Mean [SD], (95% CI)	p value[a]	Mean [SD]	Mean [SD], (95% CI)	p value[a]	Mean [SD]	Mean [SD], (95% CI)	p value[a]
**Sleeve Gastrectomy**										
	n=144	n=136			n=136			n=120		
Weight (kg)	140.0 [25.3]	117.3 [22.7]	22.8 [7.3], (21.6, 24.1)	<0.001	106.6 [21.3]	32.7 [10.7], (30.8, 34.4)	<0.001	99.2 [20.2]	39.3 [14.7], (36.6, 41.9)	<0.001
BMI (kg/m^2)	49.7 [7.4]	41.6 [6.9]	8.0 [2.4], (7.6, 8.4)	<0.001	37.9 [6.7]	11.5 [3.5], (10.9, 12.1)	<0.001	35.1 [6.1]	13.9 [5.0], (13.0, 14.8)	<0.001
%TWL	n/a	12.6 [3.8]	n/a	n/a	20.1 [5.8]	7.6 [4.5], (6.8, 8.3)	<0.001	24.8 [8.8]	12.2 [7.4], (10.8, 13.5)	<0.001
%EWL	n/a	28.2 [10.4]	n/a	n/a	45.1 [16.3]	16.9 [10.8], (15.1, 18.8)	<0.001	55.6 [20.5]	26.8 [15.7], (23.9, 29.6)	<0.001
**Gastric Bypass**										
	n=66	n=60			n=58			n=51		
Weight (kg)	137.8 [25.5]	115.7 [21.1]	19.9 [6.7], (18.1, 21.6)	<0.001	103.1 [21.4]	36.2 [10.1], (33.5, 38.8)	<0.001	93.0 [16.5]	41.6 [12.9], (38.0, 45.2)	<0.001
BMI (kg/m^2)	48.7 [8.8]	41.3 [7.8]	6.7 [3.1], (5.9, 7.5)	<0.001	36.4 [7.2]	12.8 [3.5], (11.8, 13.7)	<0.001	33.1 [5.8]	14.9 [5.0], (13.5, 16.3)	<0.001
%TWL	n/a	9.6 [3.7]	n/a	n/a	21.8 [5.5]	12.2 [4.9], (10.9, 13.5)	<0.001	26.6 [7.5]	17.1 [7.3], (15.0, 19.2)	<0.001
%EWL	n/a	23.8 [12.6]	n/a	n/a	54.0 [30.2]	31.6 [23.8], (25.0, 38.1)	<0.001	66.6 [32.5]	43.1 [26.5], (35.5, 50.7)	<0.001

[a]using the paired t-test; BMI: Body Mass Index; %TWL: percentage of total weight loss; %EWL: percentage of excess weight loss

Supplementary materials 9: Treatment of deficiency

30-50% of nutrient deficiencies, across all postoperative timepoints, were treated with multivitamin prescription, due to 88% of cases occurring in a setting where participants were non-adherent to prophylactic micronutrient supplementation. However, additional micronutrient supplement treatments required, including for vitamin D (up to 31% of participants across the three postoperative timepoints), oral iron (up to 5% of participants), IV iron (<1%), IM vitamin B12 (up to 3%), vitamin A (<1%), zinc (<1%) and selenium supplementation (<1%). Of all deficiencies, 20% were found to be persistent, as identified as occurring at two or more consecutive occasions. 91% of these persistent deficiencies occurred in a setting where participants were non-adherent to prophylactic micronutrient supplementation recommendations.
